# Supplementary material for: Low-Dose Nicotine Activates EGFR Signaling via α5-nAChR and Promotes Lung Adenocarcinoma Progression
Source: Int J Mol Sci. 2020 Sep 17;21(18):6829. doi: 10.3390/ijms21186829 (PMC7555382; doi:10.3390/ijms21186829)
Supplement: Supplementary file 1 [file ijms-21-06829-s001.zip › ijms-878148-supplementary-for publishing.docx]

# SUPPLEMENTARY INFORMATION

## SUPPLEMENTARY MATERIALS AND METHODS

## Virus generation and infection

Virus generation and infection were performed as described [1]. Briefly, HEK293T cells (2.2×10^6^) were seeded onto a 100-mm culture dish the day before transfection. Cells were cotransfected with the pLKO.1-based lentiviral vector, Δ8.9 plasmid, and vesicular stomatitis virus G protein plasmid. The medium was replaced with normal culture medium 24 h later, and the virus-containing medium was collected after another 24 h. Lentiviral infection was performed by adding the virus-containing medium to cells at the desired multiplicity of infection in the presence of 8 ng/mL polybrene. Puromycin (2 μg/mL) or G418 (800 μg/mL) selection was performed 24 h after infection for 2 or 7 days, respectively. The selected cells were subjected for experiments within 1 month.

## Quantitative real-time PCR

RNA was extracted from cells using TRIzol (Invitrogen) according to the manufacturer’s protocol. Extracted total RNA was treated with DNase before reverse transcribed into cDNA using the random hexamer primer with Superscript III reverse transcriptase (Invitrogen). Each cDNA was equally diluted for subsequent PCR amplification with the KAPA SYBR FAST ABI Prism 2X qPCR Master Mix (KAPA Biosystems) using StepOne Plus Real-Time PCR System (Applied Biosystems). The primers were designed using the Vector NTI software through importing the coding sequences of target genes from NCBI database. The top 10 primers were further analyzed by the open access Multiple Primer Analyzer software provided by ThermoFisher Scientific to avoid primer dimer. The picked primers were then blast with gene database in NCBK (pick primer blast) to confirm their specificity. The sequence of the primers designed to detect specific genes and the amplicon length are listed in Supplemental Table S1. The relative gene expression normalized to 18S was calculated using the 2^−ΔΔCt^ methods. The data represent the mean ± SD of three independent experiments.

## Proliferation assay

Cells were seeded at a density of 2000 cells per well in 24-well plates with 500 μL culture medium and allowed to adhere overnight. The culture medium was then replaced with the one supplemented with 0.5% serum. A WST-1 (Roche Applied Science) assay was performed according to the manufacturer’s protocol to assess the changes in relative cell quantity up to 7 days.

## Migration and invasion assays

A FluoroBlok 24-Multiwell Insert System with an 8-µm pore size polyethylene terephthalate membrane (BD Falcon) was used to test cell mobility. Each well was filled with 700 µL medium, and cell suspensions were seeded into the insert chamber at a density of 2.5×10^4^ cells in 300 µl medium. After 24 hours, the medium was removed, and the chamber was washed with 1× PBS and fixed in 100% methanol overnight. The reverse side of the membrane facing the lower chamber was stained with propidium iodide (Sigma-Aldrich) for 30 minutes, and the migratory cells were then visualized under an inverted microscope. Cell number was quantitated using ImageJ software. For the invasion assay, the membrane was coated with Matrigel (BD Biosciences, San Diego) diluted with an equal volume of serum-free medium and incubated for at least 1 hour at 37 °C before the cells were seeded. For the wound healing cell migration assay, 2 × 10^5^ cells were seeding into each silicon culture insert (Ibidi) in a 6-well cell culture plate and allowed to adhere overnight. Silicon inserts were removed and cells were washed with PBS. Each well of the 6-well plates were filled with 2 mL of culture medium and the migratory cells were imaged with an inverted microscope.

## Colony formation assay

Cells were seeded as single-cell in 6-well plate at a density of 500 cells per well and allowed to attach overnight. Cells were maintained in a medium containing either 10% or 0.5% serum with or without 10 nM nicotine for 14 to 21 days until visible colonies formed. Colonies were fixed with methanol and stained with crystal violet (Thermo Fisher, Pittsburgh, PA, USA) for 2 h, after which the number of colonies was counted or photomicrographs were taken under phase-contrast microscope.

## EGFR mutation analysis

Genomic DNA of independent cores was extracted from variable stage tissue microarray using proteinase K digestion which was described as previously [3]. The proteinase K digested extract is adequate for molecular tests. TaqMan EGFR Exon 19 Deletions Assay, L858R Mutation Detection Assay and Reference Assay (Invitrogen Life Technologies Inc.,) were carried out for EGFR mutation analysis according to the manufacturer’s instruction. The mutation status of a sample was determined by using the parameter described previously [4]. Samples were considered as mutation positive if a specific sigmoid curve was observed with Ct of mutation reaction ≤ 37 and/or ΔCt value (Ct of control reaction – Ct mutation reaction) ≤7. Genomic DNA of cases with inadequate tumor cells on the tissue microarrays (tumor % < 10%) were extracted from tumor whole sections and subjected to the EGFR mutation analysis described above.

## SUPPLEMENTARY FIGURES

##
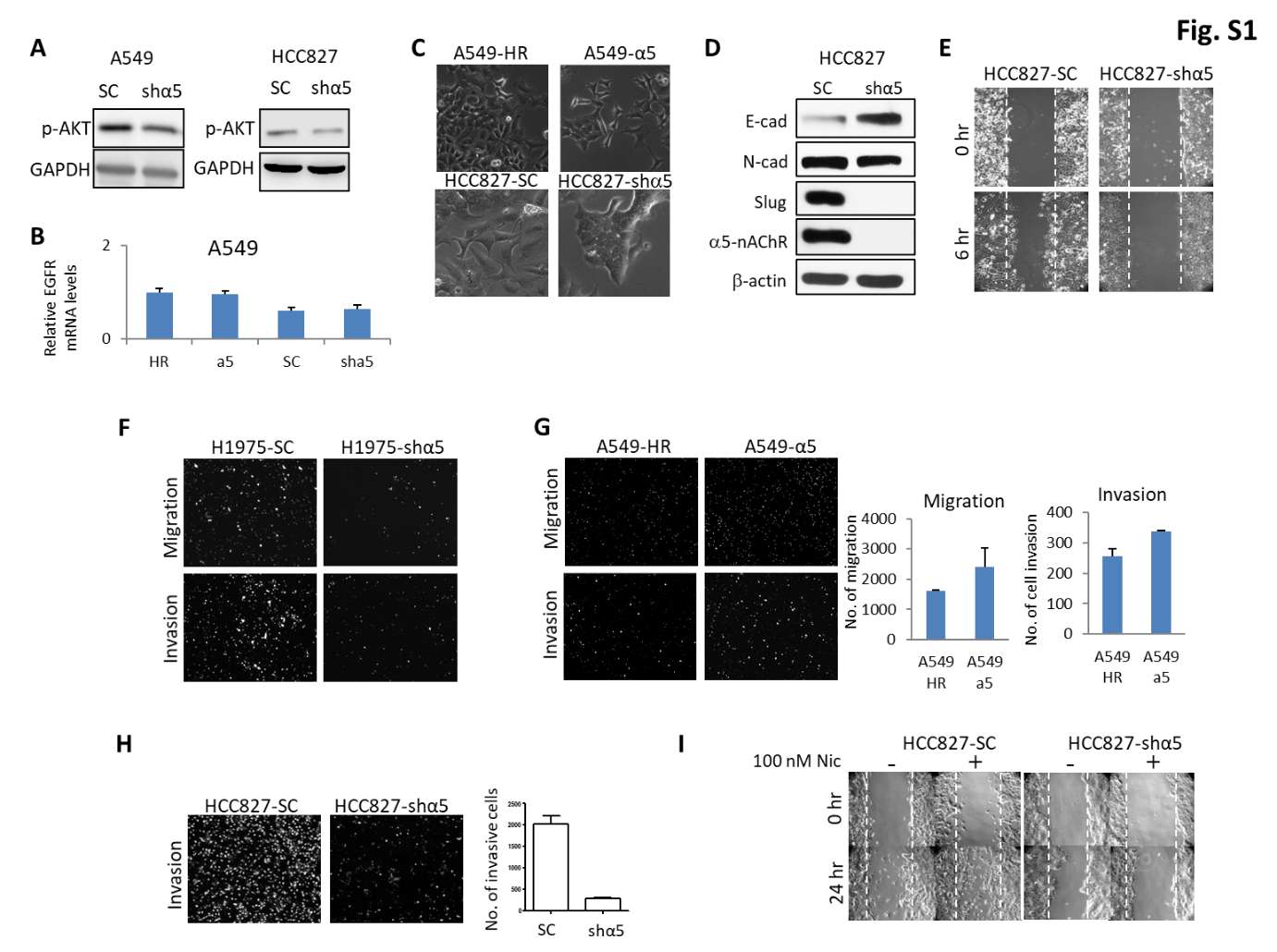


## Figure S1. Evaluation of α5-nAChR-overexpressing and knockdown stable cells lines, and the nicotine effect on EGFR phosphorylation in LAC cells. A. Knockdown of α5-nAChR in A549 and HCC827 cells suppressed the level of phosphor-Akt. B. A549 stable cells were analyzed by qRT-PCR to evaluate the mRNA expression level of α5-nAChR. C. Micrographs showing the morphology of LAC cells stably overexpressing or knockdown of α5-nAChR and their respective controls. D. Western blot analysis of EMT-related protein expression in HCC827 stable cells. E. HCC827 stable cells were subjected to a wound healing migration assay. Knockdown of α5-nAChR blocked the migration of HCC827 cells. F. H1975 stable cells were subjected to TransWell migration and invasion assay. Knockdown of α5-nAChR blocked the H1975 cell motility in both assays. G. A549 stable cells were subjected to TransWell migration and invasion assay. Knockdown of α5-nAChR blocked the A549 cell motility in both assays. Quantitative data are presented in the chart on the right. H. TransWell invasion assay of HCC827 stable cells. Knockdown of α5-nAChR inhibited the number of invasive cells. I. Control (HCC827-SC) and α5-nAChR-knockdown (HCC827-shα5) cells were subjected to a wound healing mobility assay in the presence or absence of 100 nM nicotine for 24 h.


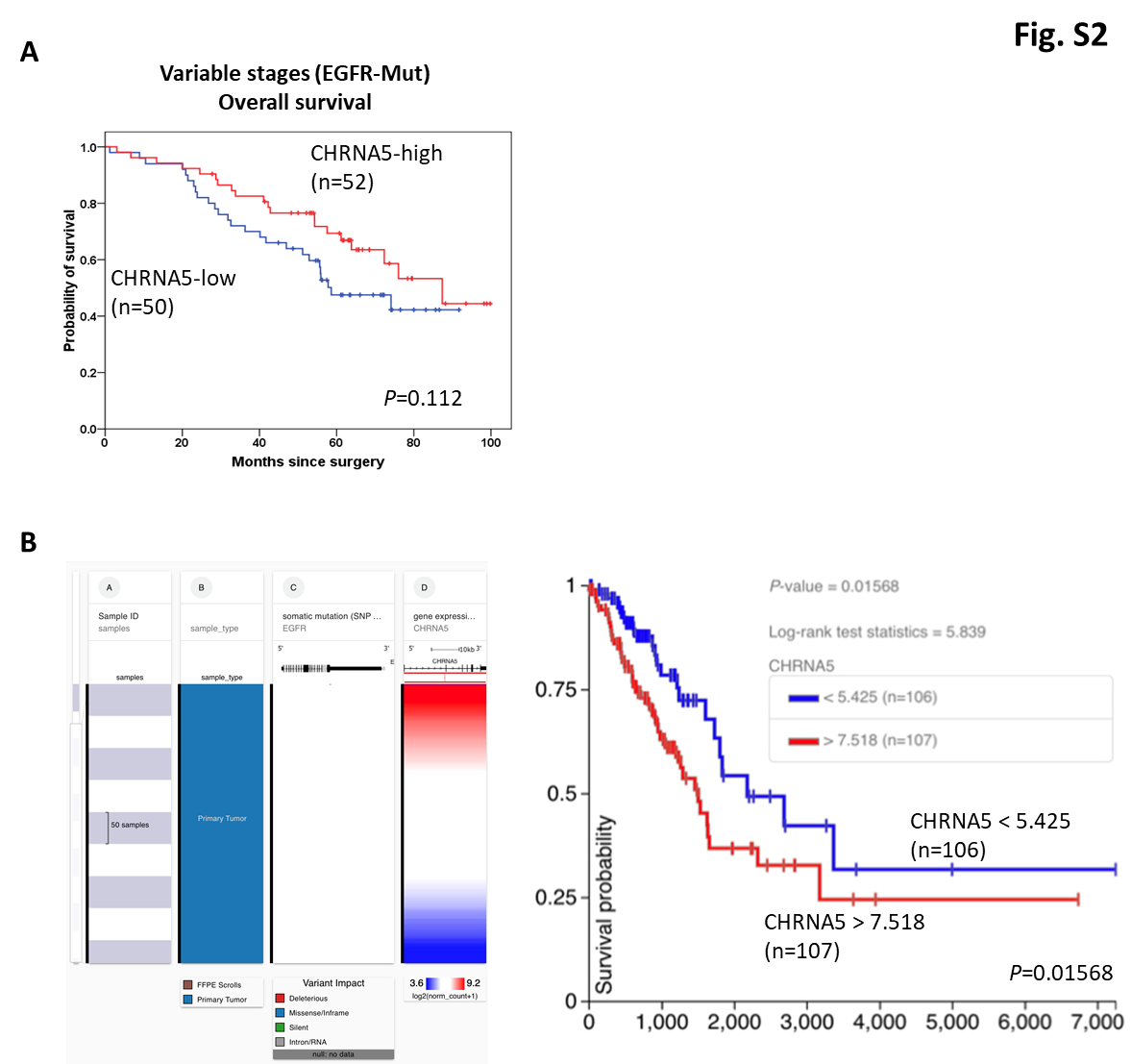


## Figure S2. Survival analysis of LAC patients. A. Kaplan-Meier analysis of the overall survival in patients with EGFR-mutant LAC at variable stages. *P* = 0.112. B. Kaplan-Meier analysis of the overall survival according to CHRNA5 mRNA expression levels in EGFR-wildtype lung adenocarcinoma patients from TCGA database (CHRNA5 > 7.518, *n* = 107 v.s. CHRNA5 < 5.425, *n* = 106). *p* = 0.01568.


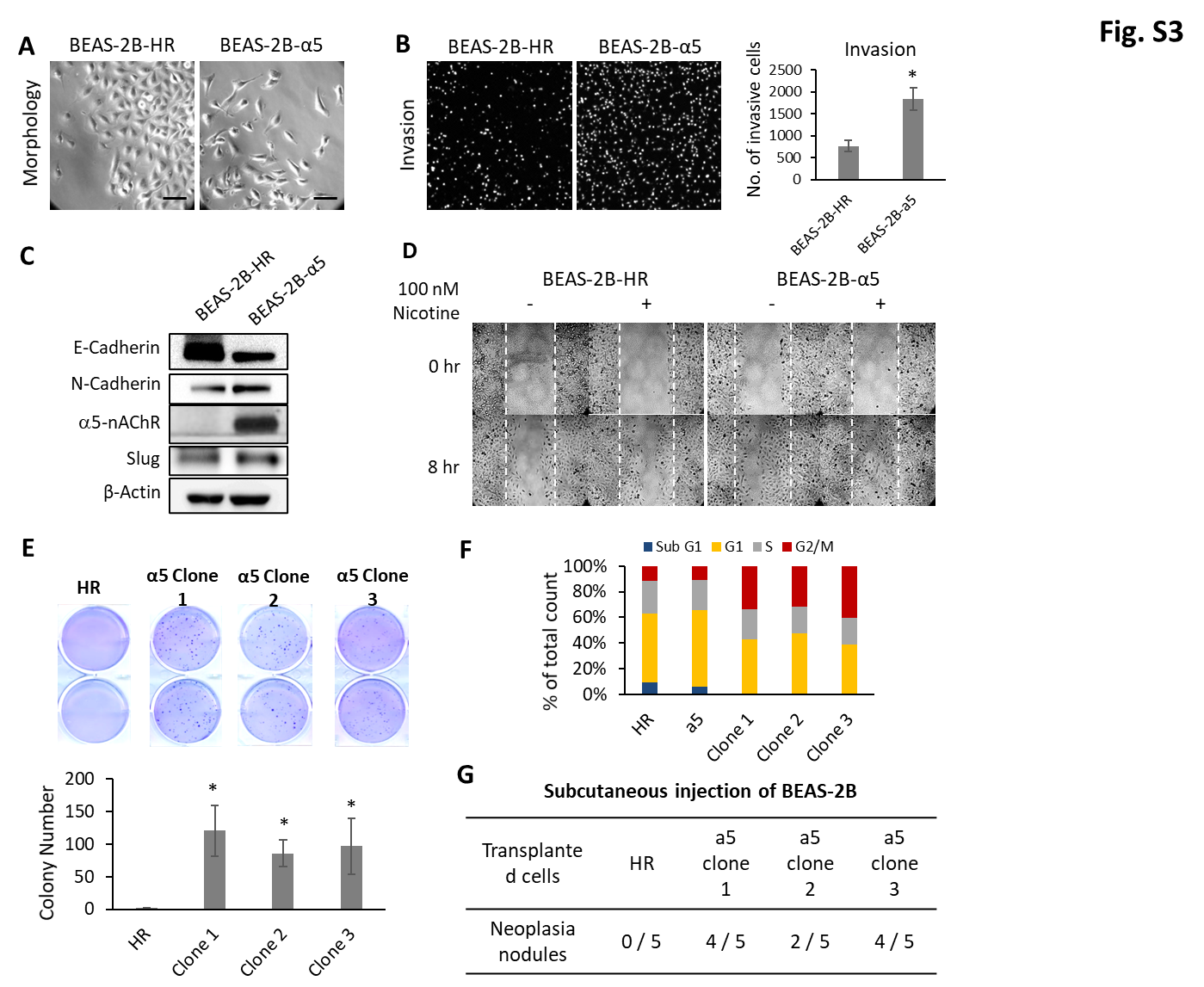


## Figure S3. α5-nAChR-dependent enhancement of cell motility and proliferation in BEAS-2B human bronchial epithelial cells. A. Micrographs showing the morphology of BEAS-2B cells stably transfected with α5-nAChR (BEAS-2B-α5) and empty vector (BEAS-2B-HR). Scale bar = 100 μm. B. BEAS-2B-HR and BEAS-2B-α5 were subjected to a Transwell invasion assay for 24 h., and the invasive cell numbers were quantified and shown to the right. C. Western blot analysis of BEAS-2B-HR and BEAS-2B-α5 cells to assess the expression levels of the indicated proteins. D. BEAS-2B-HR and BEAS-2B-α5 were subjected to a wound healing migration assay in the presence or absence of 10 nM nicotine up to 8 hours. E. BEAS-2B-HR and three clones of BEAS-2B-α5 cells were seeded in 6-well plates at a density of 500 cells per well for colony formation assays in medium supplimented with 0.5% serum for 30 days. F. BEAS-2B-HR and BEAS-2B-α5 cells were analyzed by FACS to assess the percentage of cell in each cell cycle stage. G. BEAS-2B-HR and BEAS-2B-α5 cells were subcutaneously transplanted in 4-week-old female BALB/c-nu/nu mice and monitored for up to 8 weeks (*n* = 5). The number of mice that showed hypertrophic nodules are presented in the chart.


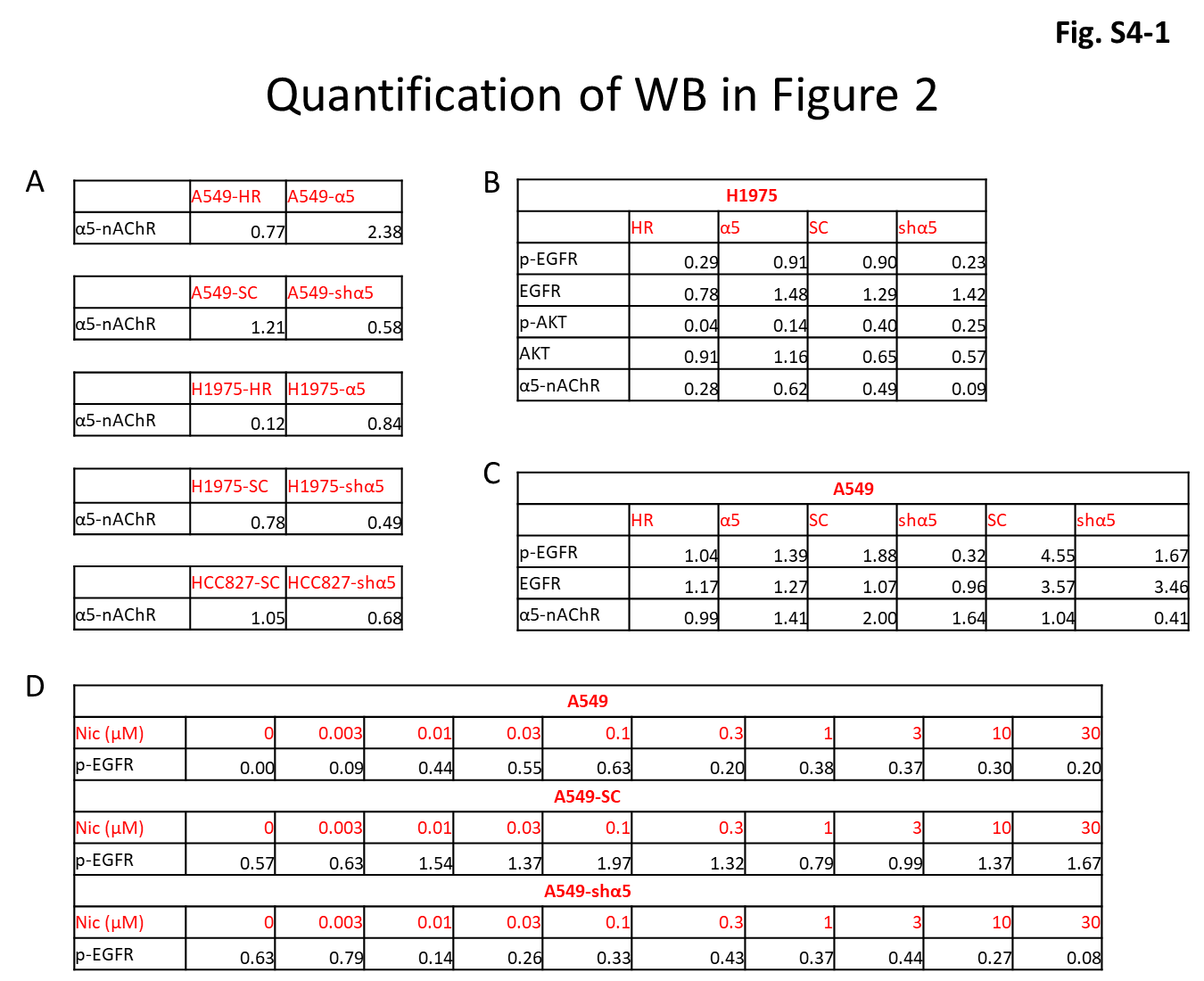


##
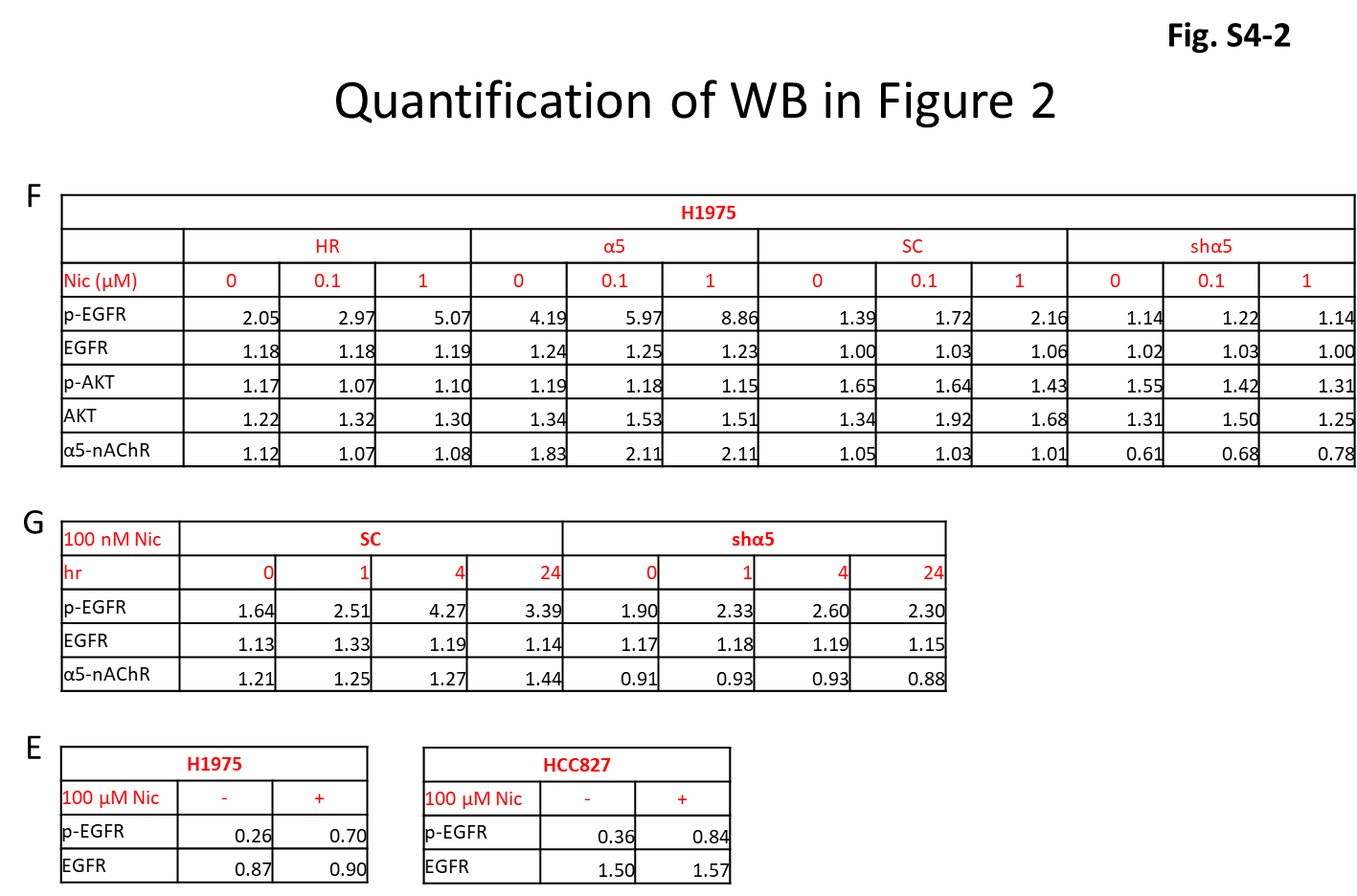


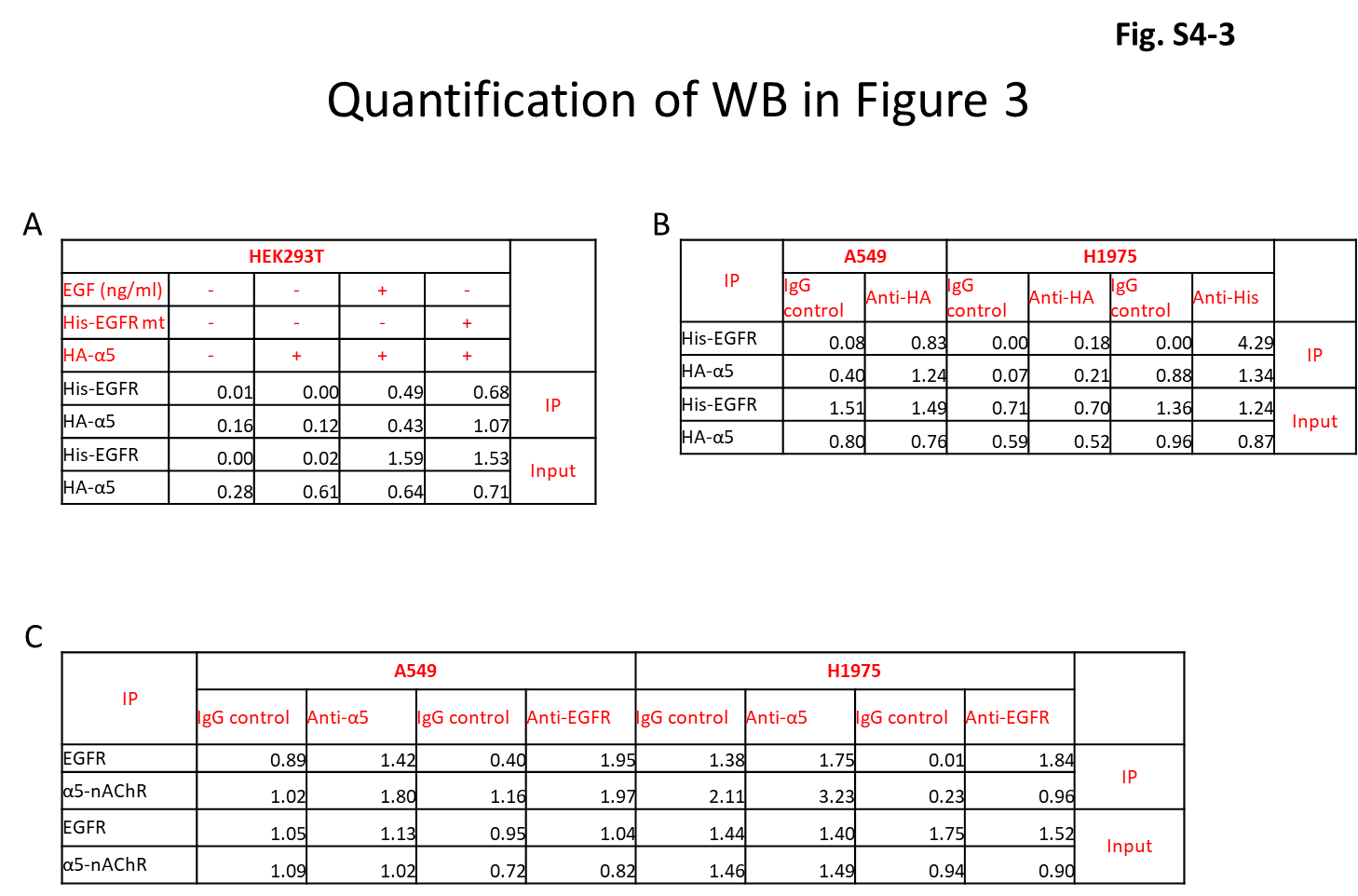


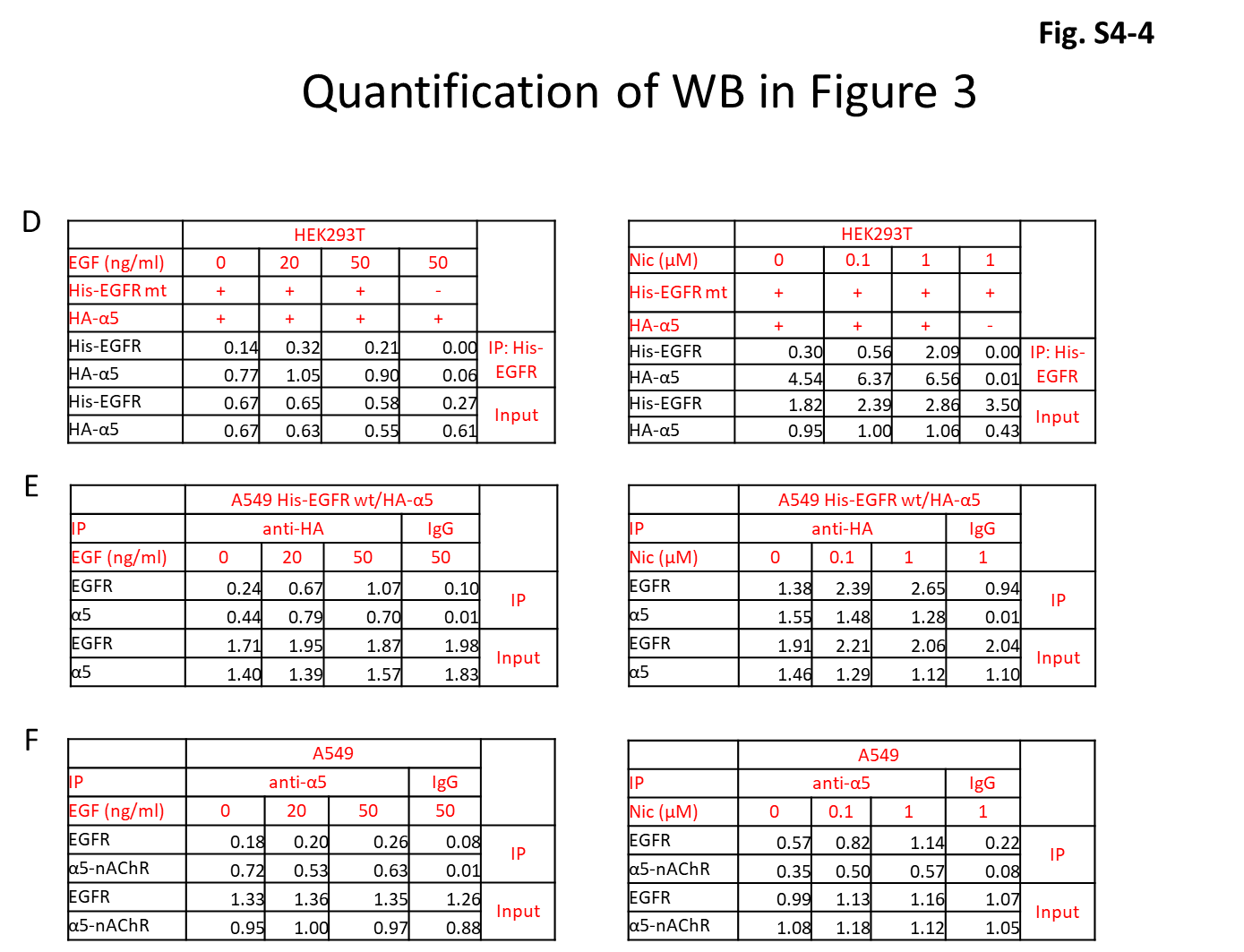


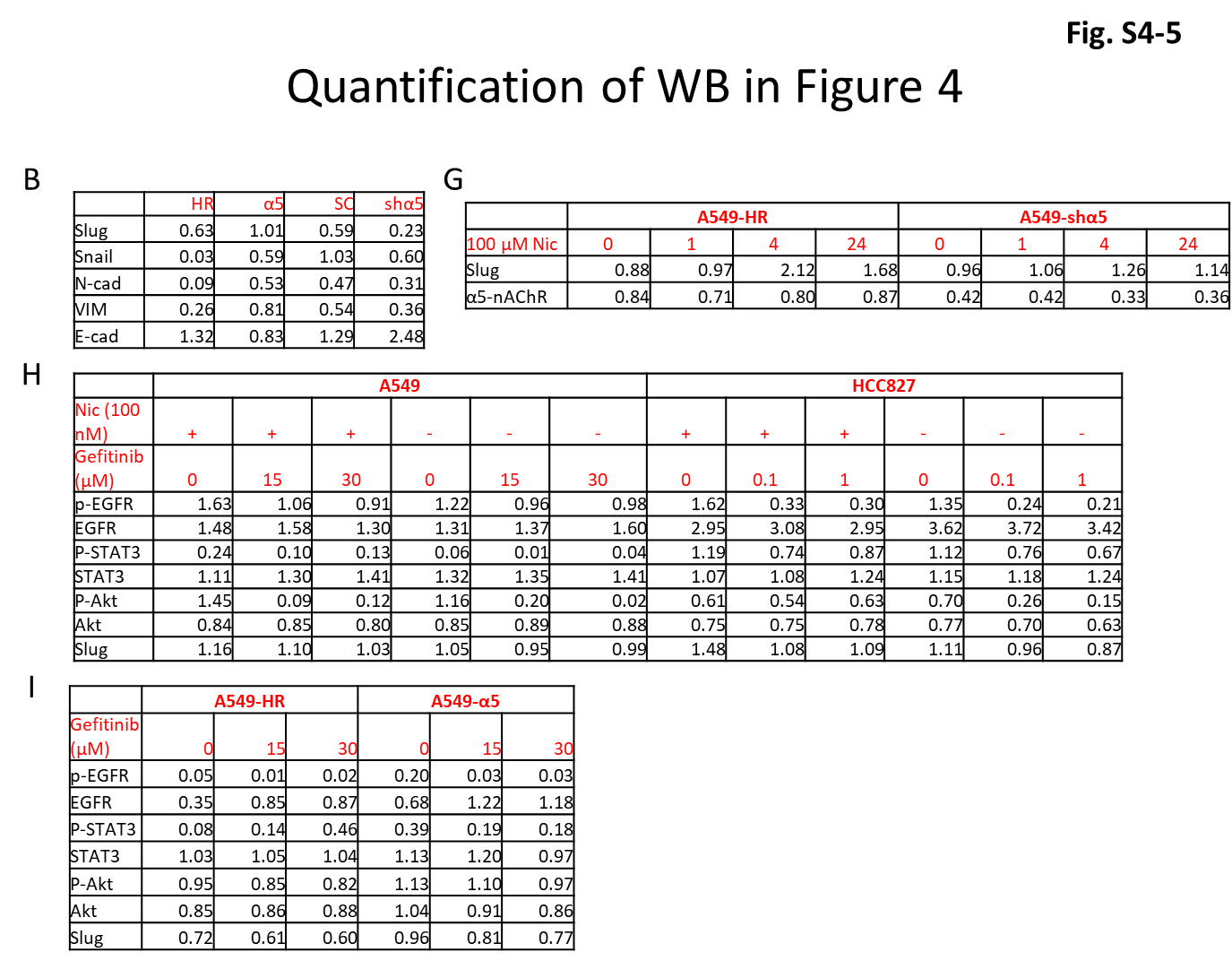


## Figure S4. Quantification of the Western Blots in Figure 2, 3, and 4. Band intensity was quantified by Image J software. The expression of each target proteins was normalized by the housekeeping genes and presented as the relative intensities to the housekeeping genes (loading control), respectively.

## SUPPLEMENTARY TABLE

**Table S1.** List of qRT-PCR primers.

| **Gene Name** | **Primer Sequence** | | **Amplicon**  **Length** | **Location (Exon)** |
| --- | --- | --- | --- | --- |
| EGFR | forward | 5’-ACTCATGCTCTACAACCC-3’ | 231 | 6,7,8 |
|  | reverse | 5’-CCAATACCTATTCCGTTACAC-3’ |  |  |
| CHRNA2 | forward | 5’- AGACCTCCCCCCAACTCTGT-3’ | 69 | 7 |
|  | reverse | 5’- ACCCGATGCCCACATCTG-3’ |  |  |
| CHRNA3 | forward | 5’’- CCCCATGGCCTAATCACCTT-3’ | 62 | 6 |
|  | reverse | 5’- ATTCCCAGCGTGATTGTCTTAGAT-3’ |  |  |
| CHRNA4 | forward | 5’- GGCGGCTTCGGATTTCTAA-3’ | 66 | 6 |
|  | reverse | 5’- GCGCCTGATCCAGCATTT-3’ |  |  |
| CHRNA5 | forward | 5’- AAATTAAGATGGAACCCTGATGAC -3’ | 123 | 4,5 |
|  | reverse | 5’- ACTGGTCCCTTCAAAACGTC -3’ |  |  |
| CHRNA6 | forward | 5’- CCAGTGGATGCTGTTTCTCAGTATTA -3’ | 72 | 5 |
|  | reverse | 5’- CAAATGTTGCATGGTCCAATTC -3’ |  |  |
| CHRNA7 | forward | 5’- CAGATTCCGGGGAGAAGATTTC -3’ | 93 | 7,8 |
|  | reverse | 5’- ATGTTGCGGGCATGATCTCA-3’ |  |  |
| CHRNA9 | forward | 5’- ATCCCATGCGTCCTCATATC-3’ | 60 | 4 |
|  | reverse | 5’- GGAGGCTGCTGGGAGATAA-3’ |  |  |
| CHRNA10 | forward | 5’- TGCCACTCCCCTAATTCCAA-3’ | 71 | 5 |
|  | reverse | 5’- CCTCCAAGACTGTACTCTGTGATCTT-3’ |  |  |
| 18S RNA | forward | 5’-GGCGGCGTTATTCCCATGA-3’ | 161 | 1 |
|  | reverse | 5’-GAGGTTTCCCGTGTTGAG-3’ |  |  |

**Table S2.** List of antibodies used in this study**.**

| **Antibodies** | **Protein Size (kDa)** | **Working Dilution** | **Supplier** | **Catalog Number** |
| --- | --- | --- | --- | --- |
| AKT | 60 | 1:1000 (WB) | Cell Signaling | 4685 |
| Alpha-Tubulin | 50 | 1:5000 (WB) | Sigma | T6074 |
| Anti-HA | N/A | 1:2000 (WB)  1:100 (IP) | BioLegend | 901501 |
| Beta-actin | 42 | 1:5000 (WB) | Sigma | A5441 |
| CHRNA5 | 50 ~ 53 | 1:500 (WB)  1:200 (IP) | Abnova | H00001138-M01 |
| E-cadherin | 135 | 1:1000 (WB) | Cell Signaling | 3195 |
| GAPDH | 37 | 1:5000 (WB) | Sigma | G8795 |
| EGFR | 175 | 1:1000 (WB)  1:100 (IP) | Cell Signaling | 4267 |
| His-tag | N/A | 1:1000 (WB)  1:25(IP) | Cell Signaling | 2365 |
| N-cadherin | 140 | 1:1000 (WB) | Cell Signaling | 4061 |
| Phospho-AKT  (Ser473) | 60 | 1:1000 (WB) | Cell Signaling | 4060 |
| Phospho-EGFR  (Y1068) | 175 | 1:1000 (WB) | Cell Signaling | 3777 |
| Phospho-STAT3  (Tyr705) | 79, 86 | 1:1000 (WB) | Cell Signaling | 9131 |
| Slug | 30 | 1:1000 (WB) | Cell Signaling | 9585 |
| Snail | 29 | 1:1000 (WB) | Cell Signaling | 3895 |
| STAT3 | 79, 86 | 1:1000 (WB) | Cell Signaling | 12640 |
| Viementin | 57 | 1:1000 (WB) | Thermo | MA5-11883 |
| CHRNA5 | 50 ~ 53 | 1:400 (IHC) | Lifespan | LS-C37656 |
| Anti-rabbit IgG, HRP-linked | N/A | 1:5000 (WB) | Cell Signaling | 7074 |
| Anti-mouse IgG, HRP-linked | N/A | 1:5000 (WB) | Cell Signaling | 7076 |

## SUPPLEMENTARY REFERENCE

1. H. Y. Kuo, Y. C. Chen, H. Y. Chang, J. C. Jeng, E. H. Lin, C. M. Pan, Y. W. Chang, M. L. Wang, Y. T. Chou, H. M. Shih, C. W. Wu, The PML isoform IV is a negative regulator of nuclear EGFR's transcriptional activity in lung cancer. *Carcinogenesis* **34**, 1708-1716 (2013).

2. C. F. Hong, Y. T. Chou, Y. S. Lin, C. W. Wu, MAD2B, a novel TCF4-binding protein, modulates TCF4-mediated epithelial-mesenchymal transdifferentiation. *J Biol Chem* **284**, 19613-19622 (2009).

3. H. L. Ho, F. P. Chang, H. H. Ma, L. R. Liao, Y. T. Chuang, Y. C. Chang-Chien, K. Y. Lin, T. Y. Chou, Molecular diagnostic algorithm for epidermal growth factor receptor mutation detection in Asian lung adenocarcinomas: comprehensive analyses of 445 Taiwanese patients with immunohistochemistry, PCR-direct sequencing and Scorpion/ARMS methods. *Respirology (Carlton, Vic.)* **18**, 1261-1270 (2013).

4. C. Roma, C. Esposito, A. M. Rachiglio, R. Pasquale, A. Iannaccone, N. Chicchinelli, R. Franco, R. Mancini, S. Pisconti, A. De Luca, G. Botti, A. Morabito, N. Normanno, Detection of EGFR mutations by TaqMan mutation detection assays powered by competitive allele-specific TaqMan PCR technology. *BioMed research international* **2013**, 385087 (2013).
