# Supplementary material for: Low-Dose Nicotine Activates EGFR Signaling via α5-nAChR and Promotes Lung Adenocarcinoma Progression
Source: Int J Mol Sci. 2020 Sep 17;21(18):6829. doi: 10.3390/ijms21186829 (PMC7555382; doi:10.3390/ijms21186829)

## Slide 1
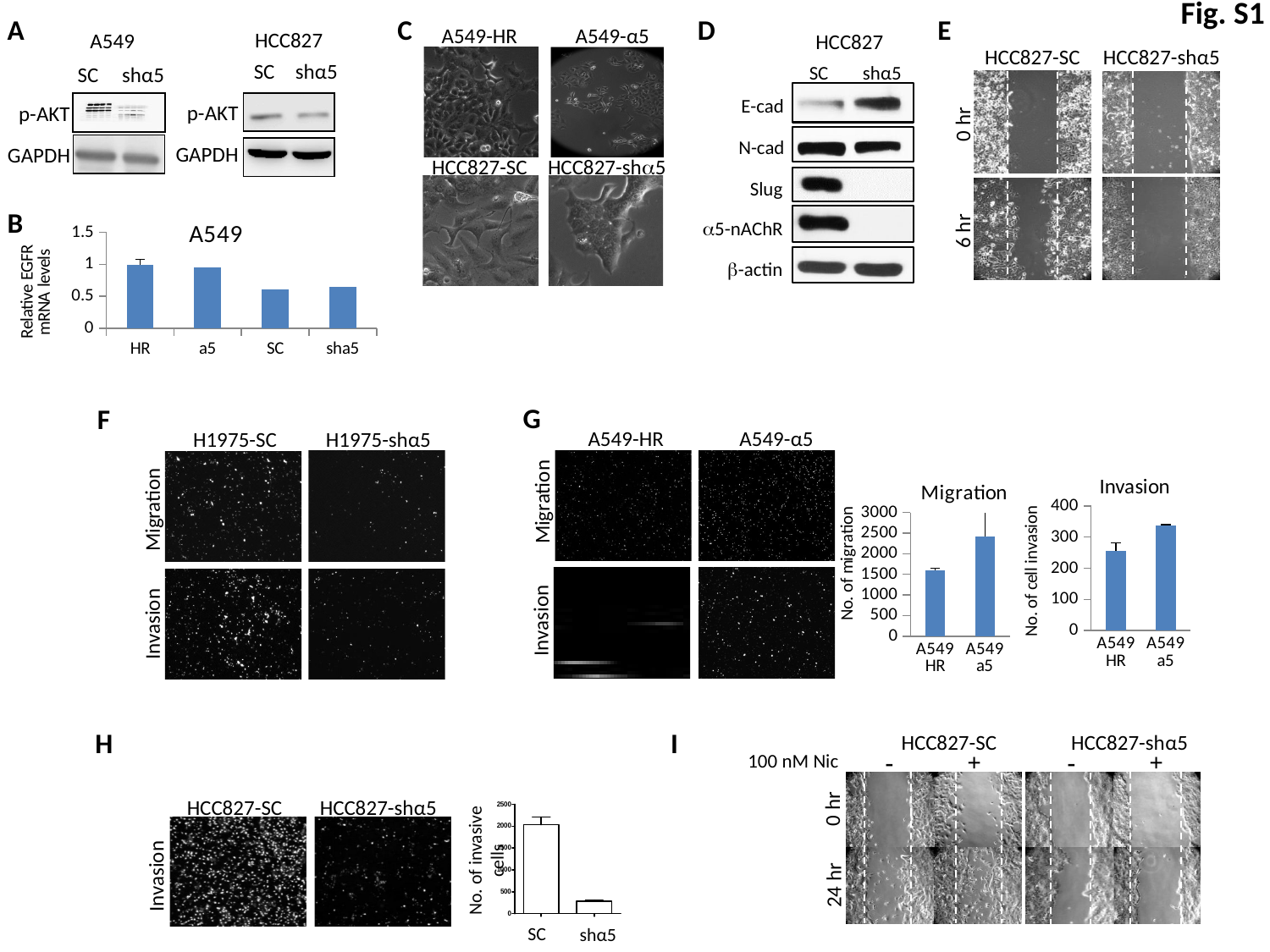

Fig. S1
A
C
D
E
A549-HR
A549-α5
HCC827-SC
HCC827-sha5
HCC827
SC
shα5
p-AKT
GAPDH
A549
SC
shα5
p-AKT
GAPDH
HCC827
SC
shα5
E-cad
N-cad
Slug
a5-nAChR
b-actin
HCC827-SC
HCC827-shα5
0 hr
6 hr
B
### Chart: A549
| Category | EGFR |
|---|---|
| HR | 1.0 |
| a5 | 0.949071392675944 |
| SC | 0.606735319173237 |
| sha5 | 0.643979023891924 |G
F
A549-HR
A549-α5
Migration
Invasion
H1975-SC
H1975-shα5
Migration
Invasion
### Chart: Invasion
| Category | |
|---|---|
| A549 HR | 255.5 |
| A549 a5 | 338.0 |
### Chart: Migration
| Category | |
|---|---|
| A549 HR | 1603.0 |
| A549 a5 | 2418.0 |H
I
HCC827-SC
HCC827-shα5
 - + - +
100 nM Nic
0 hr
24 hr
No. of invasive cells
SC
shα5
HCC827-SC
HCC827-shα5
Invasion

## Slide 2
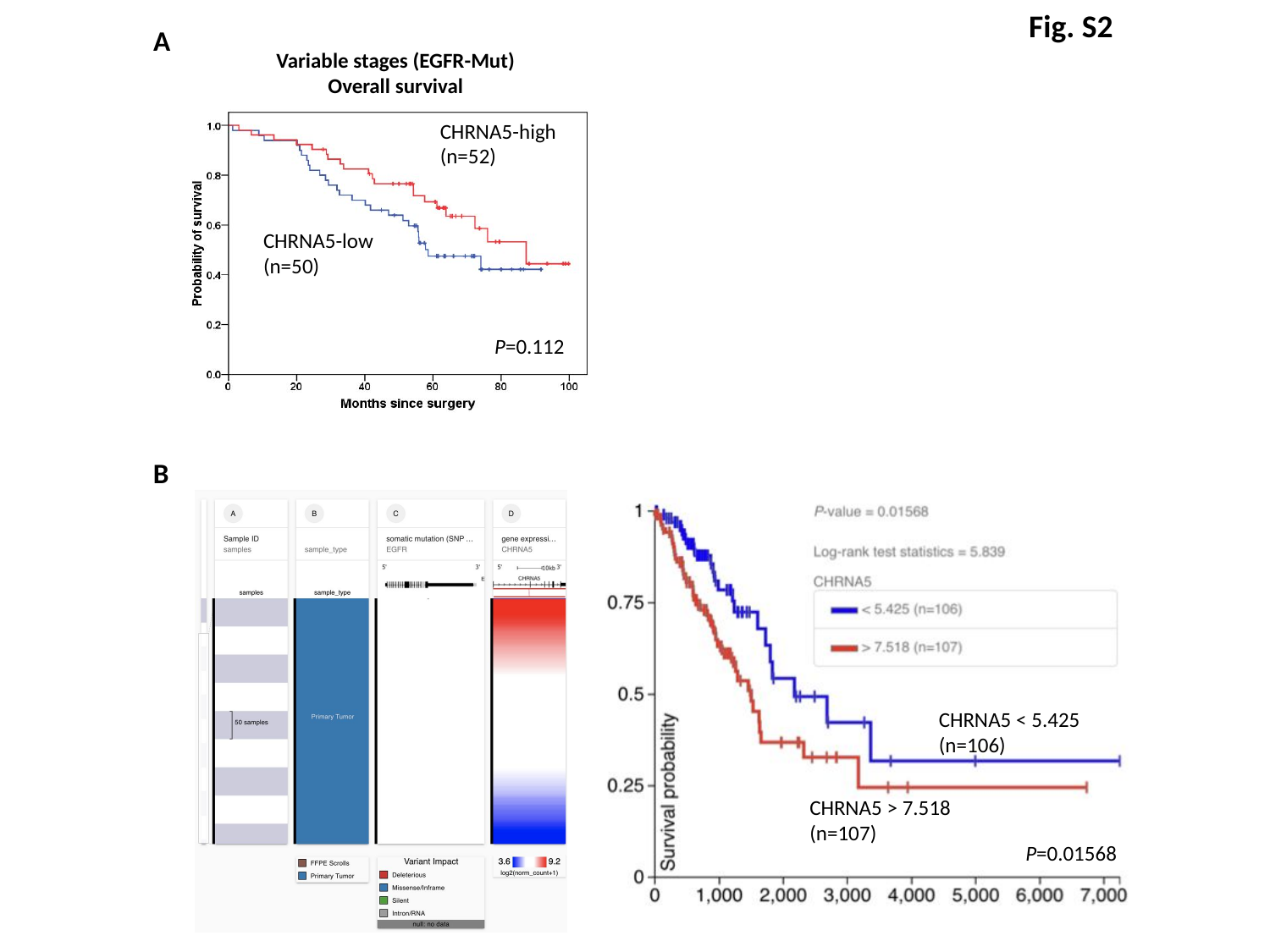

Fig. S2
A
Variable stages (EGFR-Mut)
Overall survival
CHRNA5-high
(n=52)
CHRNA5-low
(n=50)
P=0.112
B
CHRNA5 < 5.425
(n=106)
CHRNA5 > 7.518
(n=107)
P=0.01568

## Slide 3
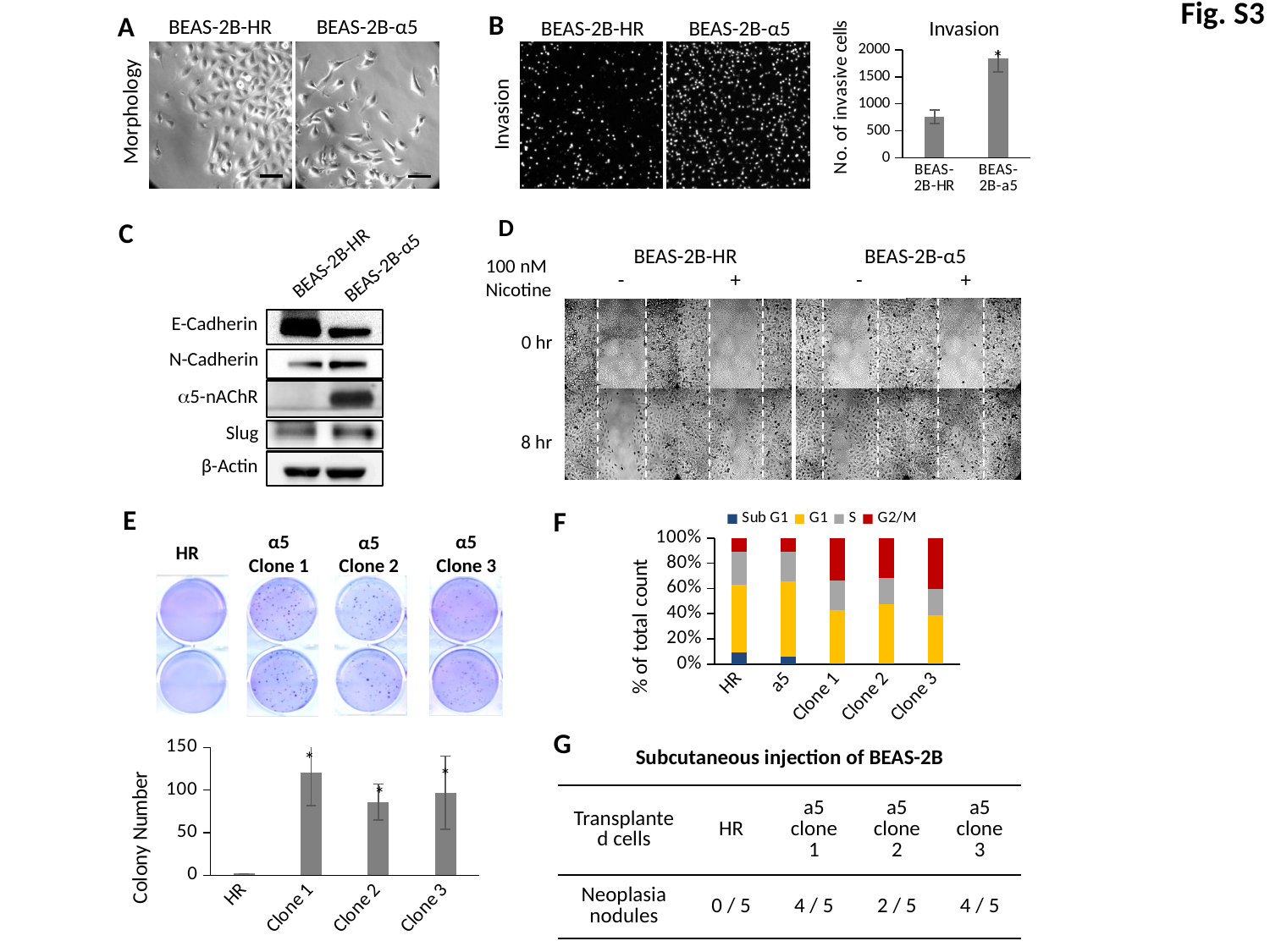

### Chart: Invasion
| Category | Cell Number |
|---|---|
| BEAS-2B-HR | 759.0 |
| BEAS-2B-a5 | 1835.0 |Fig. S3
B
A
BEAS-2B-α5
BEAS-2B-HR
Morphology
BEAS-2B-α5
BEAS-2B-HR
Invasion
*
D
C
BEAS-2B-HR
BEAS-2B-α5
E-Cadherin
N-Cadherin
a5-nAChR
Slug
β-Actin
BEAS-2B-HR
BEAS-2B-α5
100 nM
Nicotine
-
+
-
+
0 hr
8 hr
E
F
### Chart
| Category | Sub G1 | G1 | S | G2/M |
|---|---|---|---|---|
| HR | 8.32 | 48.97 | 23.55 | 10.19 |
| a5 | 5.91 | 59.59 | 24.04 | 10.65 |
| Clone 1 | 0.39 | 42.8 | 23.27 | 33.92 |
| Clone 2 | 0.36 | 47.63 | 20.29 | 32.01 |
| Clone 3 | 0.22 | 38.56 | 20.59 | 40.24 |α5 Clone 3
α5 Clone 1
α5 Clone 2
HR
G
### Chart
| Category | Colony number |
|---|---|
| HR | 1.8 |
| Clone 1 | 121.0 |
| Clone 2 | 86.0 |
| Clone 3 | 97.0 || Subcutaneous injection of BEAS-2B | | | | |
| --- | --- | --- | --- | --- |
| Transplanted cells | HR | a5 clone 1 | a5 clone 2 | a5 clone 3 |
| Neoplasia nodules | 0 / 5 | 4 / 5 | 2 / 5 | 4 / 5 |
*
*
*

## Slide 4
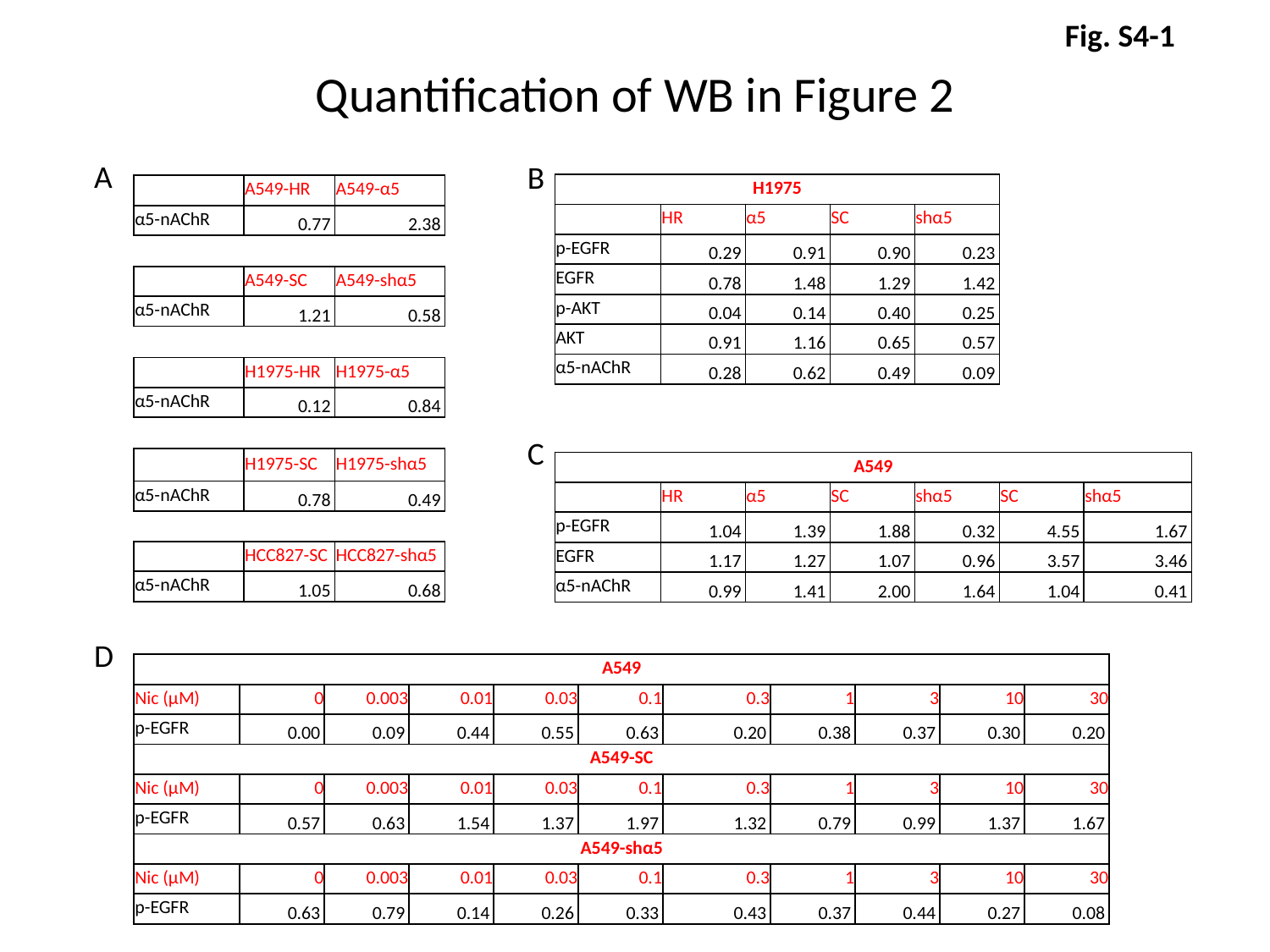

Fig. S4-1
# Quantification of WB in Figure 2
A
B
| H1975 | | | | |
| --- | --- | --- | --- | --- |
| | HR | α5 | SC | shα5 |
| p-EGFR | 0.29 | 0.91 | 0.90 | 0.23 |
| EGFR | 0.78 | 1.48 | 1.29 | 1.42 |
| p-AKT | 0.04 | 0.14 | 0.40 | 0.25 |
| AKT | 0.91 | 1.16 | 0.65 | 0.57 |
| α5-nAChR | 0.28 | 0.62 | 0.49 | 0.09 |
| | A549-HR | A549-α5 |
| --- | --- | --- |
| α5-nAChR | 0.77 | 2.38 |
| | A549-SC | A549-shα5 |
| --- | --- | --- |
| α5-nAChR | 1.21 | 0.58 |
| | H1975-HR | H1975-α5 |
| --- | --- | --- |
| α5-nAChR | 0.12 | 0.84 |
C
| | H1975-SC | H1975-shα5 |
| --- | --- | --- |
| α5-nAChR | 0.78 | 0.49 |
| A549 | | | | | | |
| --- | --- | --- | --- | --- | --- | --- |
| | HR | α5 | SC | shα5 | SC | shα5 |
| p-EGFR | 1.04 | 1.39 | 1.88 | 0.32 | 4.55 | 1.67 |
| EGFR | 1.17 | 1.27 | 1.07 | 0.96 | 3.57 | 3.46 |
| α5-nAChR | 0.99 | 1.41 | 2.00 | 1.64 | 1.04 | 0.41 |
| | HCC827-SC | HCC827-shα5 |
| --- | --- | --- |
| α5-nAChR | 1.05 | 0.68 |
D
| A549 | | | | | | | | | | |
| --- | --- | --- | --- | --- | --- | --- | --- | --- | --- | --- |
| Nic (μM) | 0 | 0.003 | 0.01 | 0.03 | 0.1 | 0.3 | 1 | 3 | 10 | 30 |
| p-EGFR | 0.00 | 0.09 | 0.44 | 0.55 | 0.63 | 0.20 | 0.38 | 0.37 | 0.30 | 0.20 |
| A549-SC | | | | | | | | | | |
| Nic (μM) | 0 | 0.003 | 0.01 | 0.03 | 0.1 | 0.3 | 1 | 3 | 10 | 30 |
| p-EGFR | 0.57 | 0.63 | 1.54 | 1.37 | 1.97 | 1.32 | 0.79 | 0.99 | 1.37 | 1.67 |
| A549-shα5 | | | | | | | | | | |
| Nic (μM) | 0 | 0.003 | 0.01 | 0.03 | 0.1 | 0.3 | 1 | 3 | 10 | 30 |
| p-EGFR | 0.63 | 0.79 | 0.14 | 0.26 | 0.33 | 0.43 | 0.37 | 0.44 | 0.27 | 0.08 |

## Slide 5
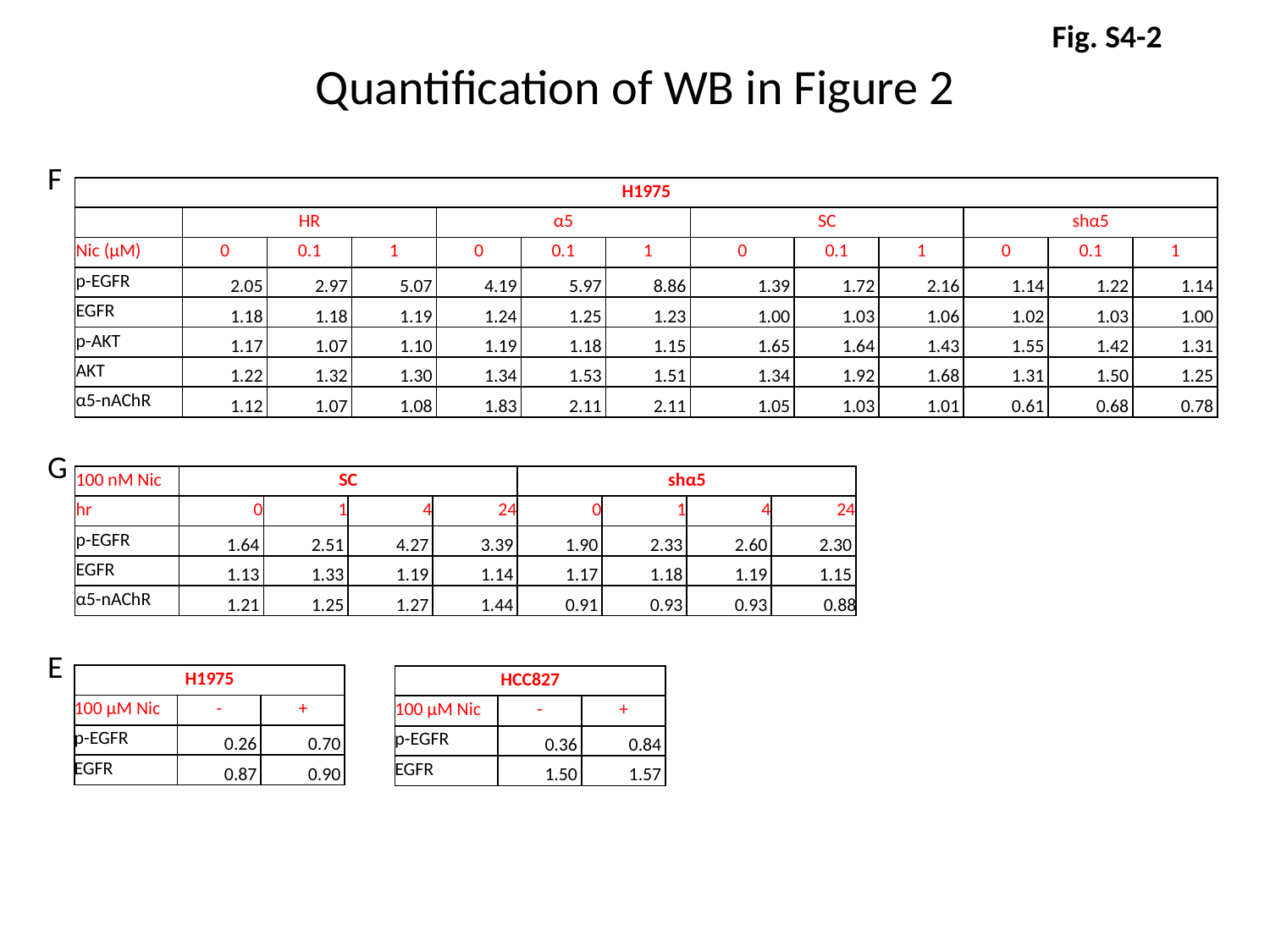

Fig. S4-2
Quantification of WB in Figure 2
F
| H1975 | | | | | | | | | | | | |
| --- | --- | --- | --- | --- | --- | --- | --- | --- | --- | --- | --- | --- |
| | HR | | | α5 | | | SC | | | shα5 | | |
| Nic (μM) | 0 | 0.1 | 1 | 0 | 0.1 | 1 | 0 | 0.1 | 1 | 0 | 0.1 | 1 |
| p-EGFR | 2.05 | 2.97 | 5.07 | 4.19 | 5.97 | 8.86 | 1.39 | 1.72 | 2.16 | 1.14 | 1.22 | 1.14 |
| EGFR | 1.18 | 1.18 | 1.19 | 1.24 | 1.25 | 1.23 | 1.00 | 1.03 | 1.06 | 1.02 | 1.03 | 1.00 |
| p-AKT | 1.17 | 1.07 | 1.10 | 1.19 | 1.18 | 1.15 | 1.65 | 1.64 | 1.43 | 1.55 | 1.42 | 1.31 |
| AKT | 1.22 | 1.32 | 1.30 | 1.34 | 1.53 | 1.51 | 1.34 | 1.92 | 1.68 | 1.31 | 1.50 | 1.25 |
| α5-nAChR | 1.12 | 1.07 | 1.08 | 1.83 | 2.11 | 2.11 | 1.05 | 1.03 | 1.01 | 0.61 | 0.68 | 0.78 |
G
| 100 nM Nic | SC | | | | shα5 | | | |
| --- | --- | --- | --- | --- | --- | --- | --- | --- |
| hr | 0 | 1 | 4 | 24 | 0 | 1 | 4 | 24 |
| p-EGFR | 1.64 | 2.51 | 4.27 | 3.39 | 1.90 | 2.33 | 2.60 | 2.30 |
| EGFR | 1.13 | 1.33 | 1.19 | 1.14 | 1.17 | 1.18 | 1.19 | 1.15 |
| α5-nAChR | 1.21 | 1.25 | 1.27 | 1.44 | 0.91 | 0.93 | 0.93 | 0.88 |
E
| H1975 | | |
| --- | --- | --- |
| 100 μM Nic | - | + |
| p-EGFR | 0.26 | 0.70 |
| EGFR | 0.87 | 0.90 |
| HCC827 | | |
| --- | --- | --- |
| 100 μM Nic | - | + |
| p-EGFR | 0.36 | 0.84 |
| EGFR | 1.50 | 1.57 |

## Slide 6
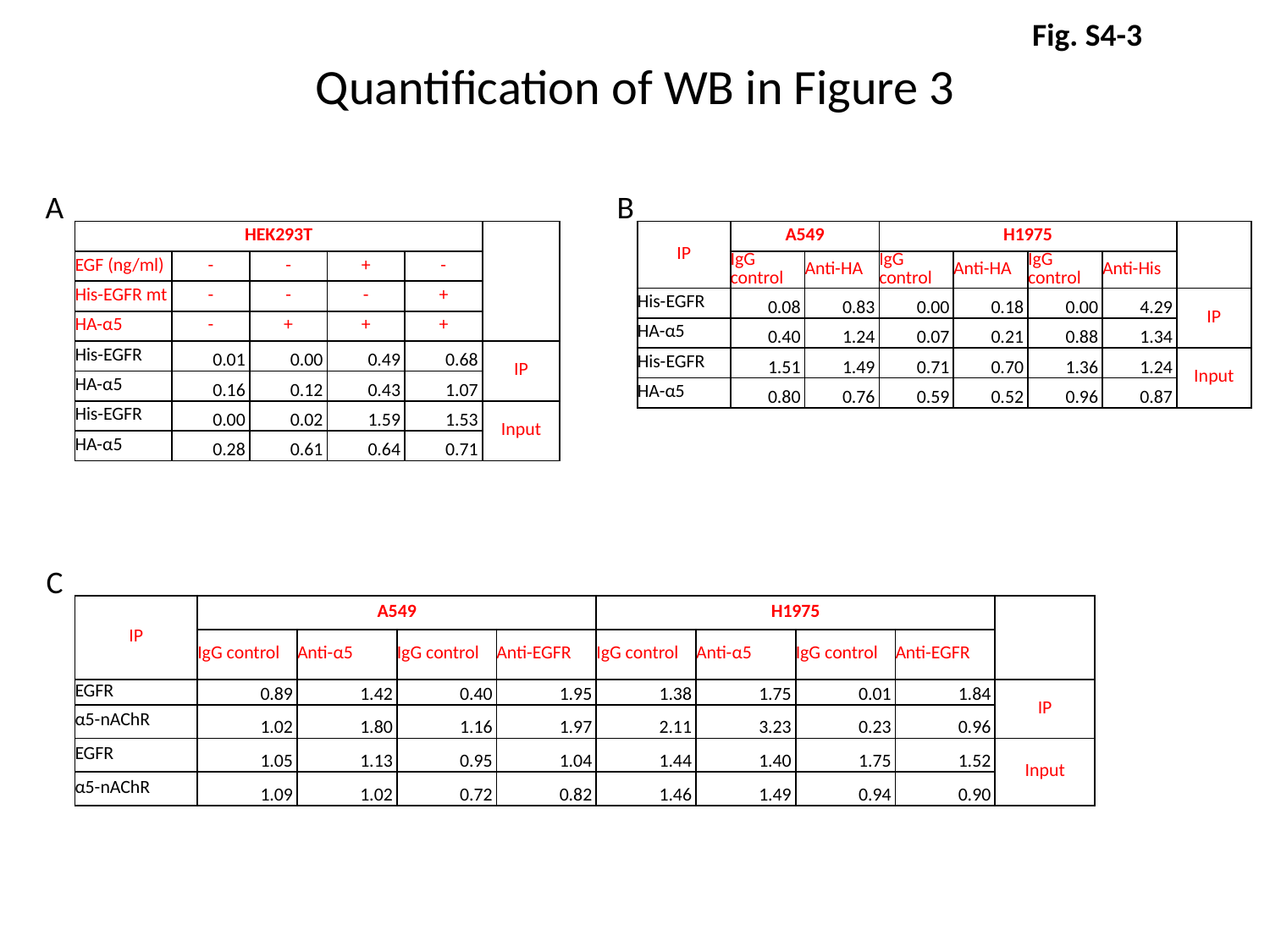

Fig. S4-3
Quantification of WB in Figure 3
A
B
| HEK293T | | | | | |
| --- | --- | --- | --- | --- | --- |
| EGF (ng/ml) | - | - | + | - | |
| His-EGFR mt | - | - | - | + | |
| HA-α5 | - | + | + | + | |
| His-EGFR | 0.01 | 0.00 | 0.49 | 0.68 | IP |
| HA-α5 | 0.16 | 0.12 | 0.43 | 1.07 | |
| His-EGFR | 0.00 | 0.02 | 1.59 | 1.53 | Input |
| HA-α5 | 0.28 | 0.61 | 0.64 | 0.71 | |
| IP | A549 | | H1975 | | | | |
| --- | --- | --- | --- | --- | --- | --- | --- |
| | IgG control | Anti-HA | IgG control | Anti-HA | IgG control | Anti-His | |
| His-EGFR | 0.08 | 0.83 | 0.00 | 0.18 | 0.00 | 4.29 | IP |
| HA-α5 | 0.40 | 1.24 | 0.07 | 0.21 | 0.88 | 1.34 | |
| His-EGFR | 1.51 | 1.49 | 0.71 | 0.70 | 1.36 | 1.24 | Input |
| HA-α5 | 0.80 | 0.76 | 0.59 | 0.52 | 0.96 | 0.87 | |
C
| IP | A549 | | | | H1975 | | | | |
| --- | --- | --- | --- | --- | --- | --- | --- | --- | --- |
| | IgG control | Anti-α5 | IgG control | Anti-EGFR | IgG control | Anti-α5 | IgG control | Anti-EGFR | |
| EGFR | 0.89 | 1.42 | 0.40 | 1.95 | 1.38 | 1.75 | 0.01 | 1.84 | IP |
| α5-nAChR | 1.02 | 1.80 | 1.16 | 1.97 | 2.11 | 3.23 | 0.23 | 0.96 | |
| EGFR | 1.05 | 1.13 | 0.95 | 1.04 | 1.44 | 1.40 | 1.75 | 1.52 | Input |
| α5-nAChR | 1.09 | 1.02 | 0.72 | 0.82 | 1.46 | 1.49 | 0.94 | 0.90 | |

## Slide 7
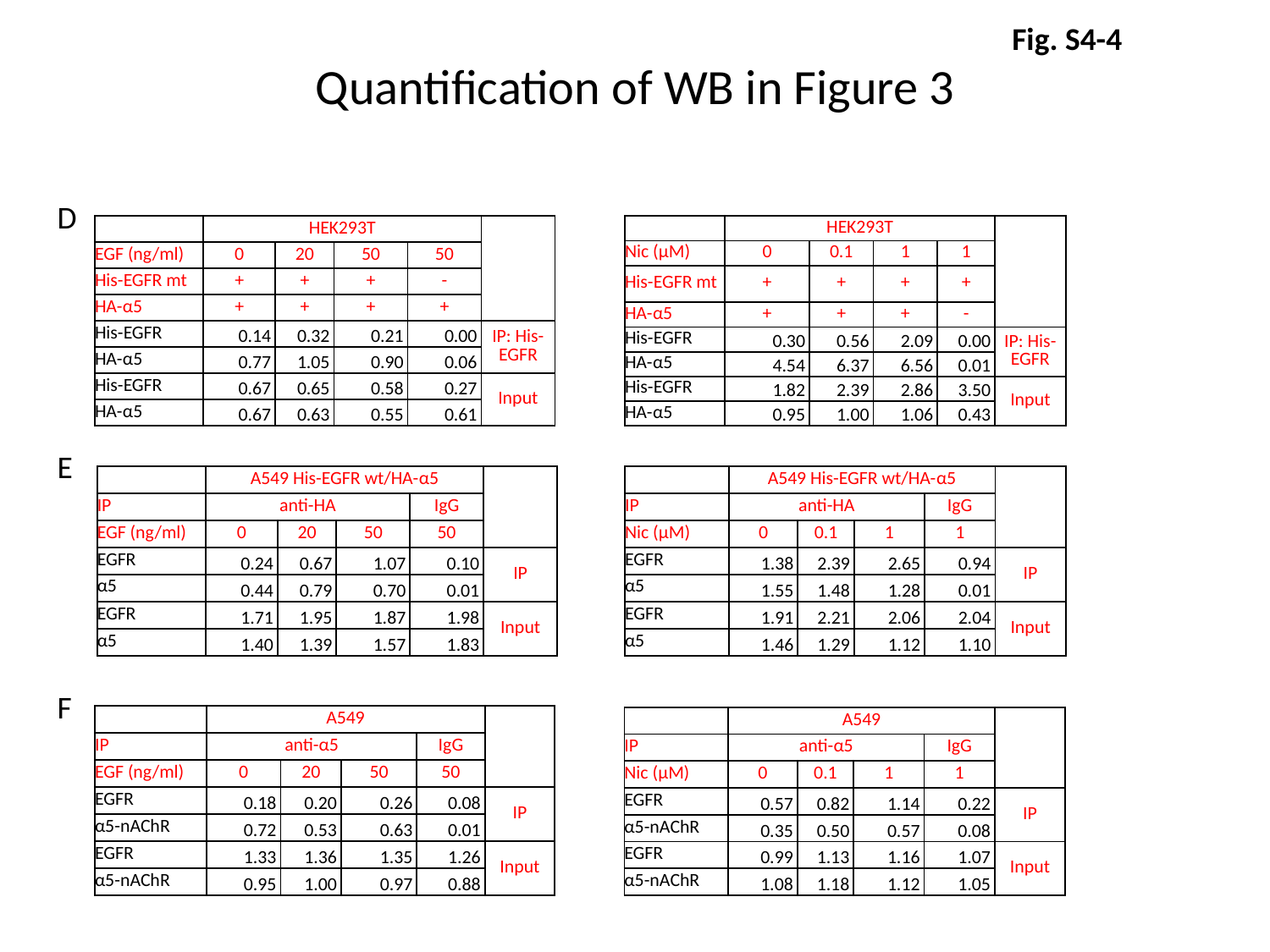

Fig. S4-4
Quantification of WB in Figure 3
D
| | HEK293T | | | | |
| --- | --- | --- | --- | --- | --- |
| EGF (ng/ml) | 0 | 20 | 50 | 50 | |
| His-EGFR mt | + | + | + | - | |
| HA-α5 | + | + | + | + | |
| His-EGFR | 0.14 | 0.32 | 0.21 | 0.00 | IP: His-EGFR |
| HA-α5 | 0.77 | 1.05 | 0.90 | 0.06 | |
| His-EGFR | 0.67 | 0.65 | 0.58 | 0.27 | Input |
| HA-α5 | 0.67 | 0.63 | 0.55 | 0.61 | |
| | HEK293T | | | | |
| --- | --- | --- | --- | --- | --- |
| Nic (μM) | 0 | 0.1 | 1 | 1 | |
| His-EGFR mt | + | + | + | + | |
| HA-α5 | + | + | + | - | |
| His-EGFR | 0.30 | 0.56 | 2.09 | 0.00 | IP: His-EGFR |
| HA-α5 | 4.54 | 6.37 | 6.56 | 0.01 | |
| His-EGFR | 1.82 | 2.39 | 2.86 | 3.50 | Input |
| HA-α5 | 0.95 | 1.00 | 1.06 | 0.43 | |
E
| | A549 His-EGFR wt/HA-α5 | | | | |
| --- | --- | --- | --- | --- | --- |
| IP | anti-HA | | | IgG | |
| EGF (ng/ml) | 0 | 20 | 50 | 50 | |
| EGFR | 0.24 | 0.67 | 1.07 | 0.10 | IP |
| α5 | 0.44 | 0.79 | 0.70 | 0.01 | |
| EGFR | 1.71 | 1.95 | 1.87 | 1.98 | Input |
| α5 | 1.40 | 1.39 | 1.57 | 1.83 | |
| | A549 His-EGFR wt/HA-α5 | | | | |
| --- | --- | --- | --- | --- | --- |
| IP | anti-HA | | | IgG | |
| Nic (μM) | 0 | 0.1 | 1 | 1 | |
| EGFR | 1.38 | 2.39 | 2.65 | 0.94 | IP |
| α5 | 1.55 | 1.48 | 1.28 | 0.01 | |
| EGFR | 1.91 | 2.21 | 2.06 | 2.04 | Input |
| α5 | 1.46 | 1.29 | 1.12 | 1.10 | |
F
| | A549 | | | | |
| --- | --- | --- | --- | --- | --- |
| IP | anti-α5 | | | IgG | |
| EGF (ng/ml) | 0 | 20 | 50 | 50 | |
| EGFR | 0.18 | 0.20 | 0.26 | 0.08 | IP |
| α5-nAChR | 0.72 | 0.53 | 0.63 | 0.01 | |
| EGFR | 1.33 | 1.36 | 1.35 | 1.26 | Input |
| α5-nAChR | 0.95 | 1.00 | 0.97 | 0.88 | |
| | A549 | | | | |
| --- | --- | --- | --- | --- | --- |
| IP | anti-α5 | | | IgG | |
| Nic (μM) | 0 | 0.1 | 1 | 1 | |
| EGFR | 0.57 | 0.82 | 1.14 | 0.22 | IP |
| α5-nAChR | 0.35 | 0.50 | 0.57 | 0.08 | |
| EGFR | 0.99 | 1.13 | 1.16 | 1.07 | Input |
| α5-nAChR | 1.08 | 1.18 | 1.12 | 1.05 | |

## Slide 8
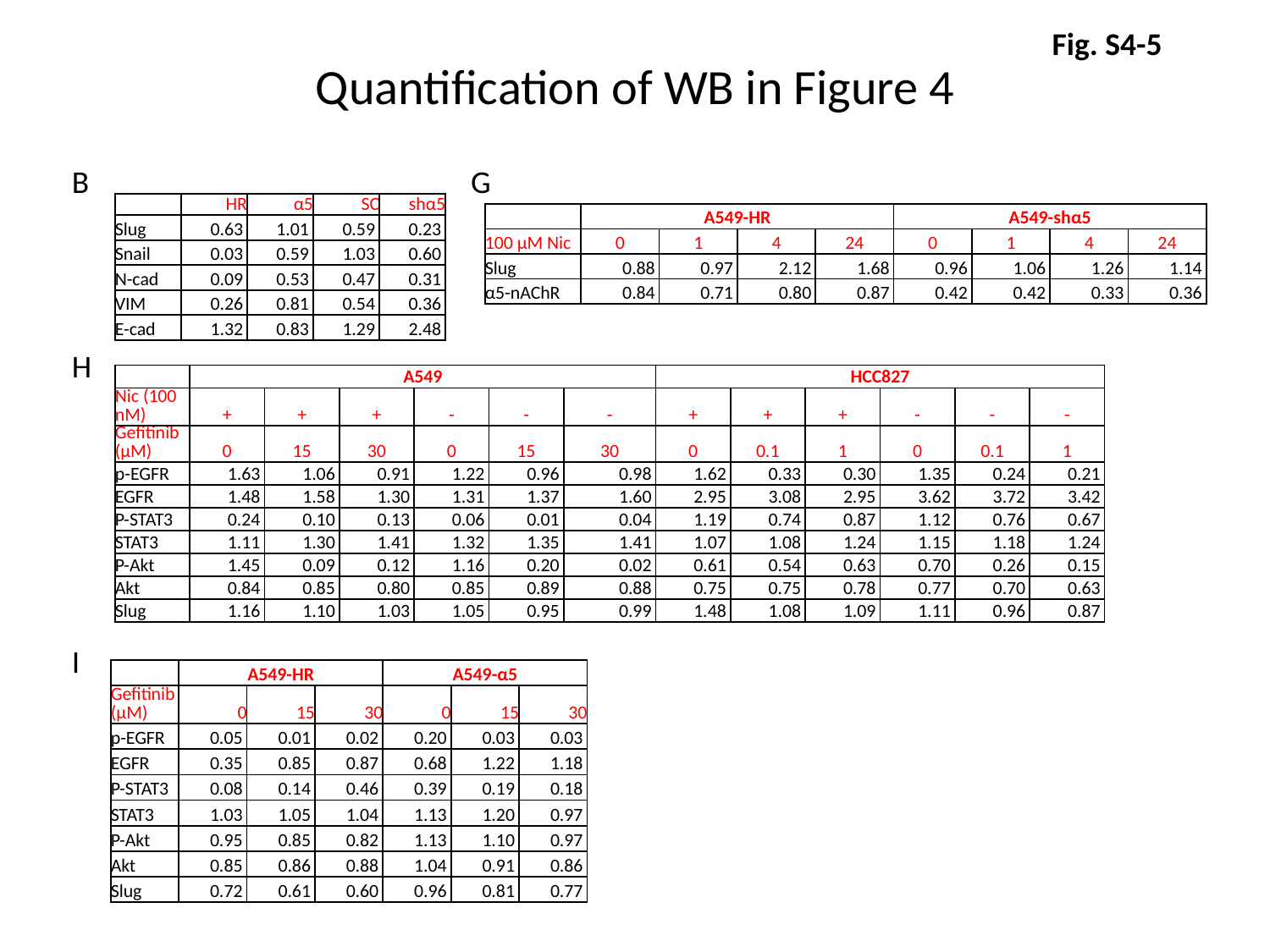

Fig. S4-5
Quantification of WB in Figure 4
B
G
| | HR | α5 | SC | shα5 |
| --- | --- | --- | --- | --- |
| Slug | 0.63 | 1.01 | 0.59 | 0.23 |
| Snail | 0.03 | 0.59 | 1.03 | 0.60 |
| N-cad | 0.09 | 0.53 | 0.47 | 0.31 |
| VIM | 0.26 | 0.81 | 0.54 | 0.36 |
| E-cad | 1.32 | 0.83 | 1.29 | 2.48 |
| | A549-HR | | | | A549-shα5 | | | |
| --- | --- | --- | --- | --- | --- | --- | --- | --- |
| 100 μM Nic | 0 | 1 | 4 | 24 | 0 | 1 | 4 | 24 |
| Slug | 0.88 | 0.97 | 2.12 | 1.68 | 0.96 | 1.06 | 1.26 | 1.14 |
| α5-nAChR | 0.84 | 0.71 | 0.80 | 0.87 | 0.42 | 0.42 | 0.33 | 0.36 |
H
| | A549 | | | | | | HCC827 | | | | | |
| --- | --- | --- | --- | --- | --- | --- | --- | --- | --- | --- | --- | --- |
| Nic (100 nM) | + | + | + | - | - | - | + | + | + | - | - | - |
| Gefitinib (μM) | 0 | 15 | 30 | 0 | 15 | 30 | 0 | 0.1 | 1 | 0 | 0.1 | 1 |
| p-EGFR | 1.63 | 1.06 | 0.91 | 1.22 | 0.96 | 0.98 | 1.62 | 0.33 | 0.30 | 1.35 | 0.24 | 0.21 |
| EGFR | 1.48 | 1.58 | 1.30 | 1.31 | 1.37 | 1.60 | 2.95 | 3.08 | 2.95 | 3.62 | 3.72 | 3.42 |
| P-STAT3 | 0.24 | 0.10 | 0.13 | 0.06 | 0.01 | 0.04 | 1.19 | 0.74 | 0.87 | 1.12 | 0.76 | 0.67 |
| STAT3 | 1.11 | 1.30 | 1.41 | 1.32 | 1.35 | 1.41 | 1.07 | 1.08 | 1.24 | 1.15 | 1.18 | 1.24 |
| P-Akt | 1.45 | 0.09 | 0.12 | 1.16 | 0.20 | 0.02 | 0.61 | 0.54 | 0.63 | 0.70 | 0.26 | 0.15 |
| Akt | 0.84 | 0.85 | 0.80 | 0.85 | 0.89 | 0.88 | 0.75 | 0.75 | 0.78 | 0.77 | 0.70 | 0.63 |
| Slug | 1.16 | 1.10 | 1.03 | 1.05 | 0.95 | 0.99 | 1.48 | 1.08 | 1.09 | 1.11 | 0.96 | 0.87 |
I
| | A549-HR | | | A549-α5 | | |
| --- | --- | --- | --- | --- | --- | --- |
| Gefitinib (μM) | 0 | 15 | 30 | 0 | 15 | 30 |
| p-EGFR | 0.05 | 0.01 | 0.02 | 0.20 | 0.03 | 0.03 |
| EGFR | 0.35 | 0.85 | 0.87 | 0.68 | 1.22 | 1.18 |
| P-STAT3 | 0.08 | 0.14 | 0.46 | 0.39 | 0.19 | 0.18 |
| STAT3 | 1.03 | 1.05 | 1.04 | 1.13 | 1.20 | 0.97 |
| P-Akt | 0.95 | 0.85 | 0.82 | 1.13 | 1.10 | 0.97 |
| Akt | 0.85 | 0.86 | 0.88 | 1.04 | 0.91 | 0.86 |
| Slug | 0.72 | 0.61 | 0.60 | 0.96 | 0.81 | 0.77 |

## Slide 9
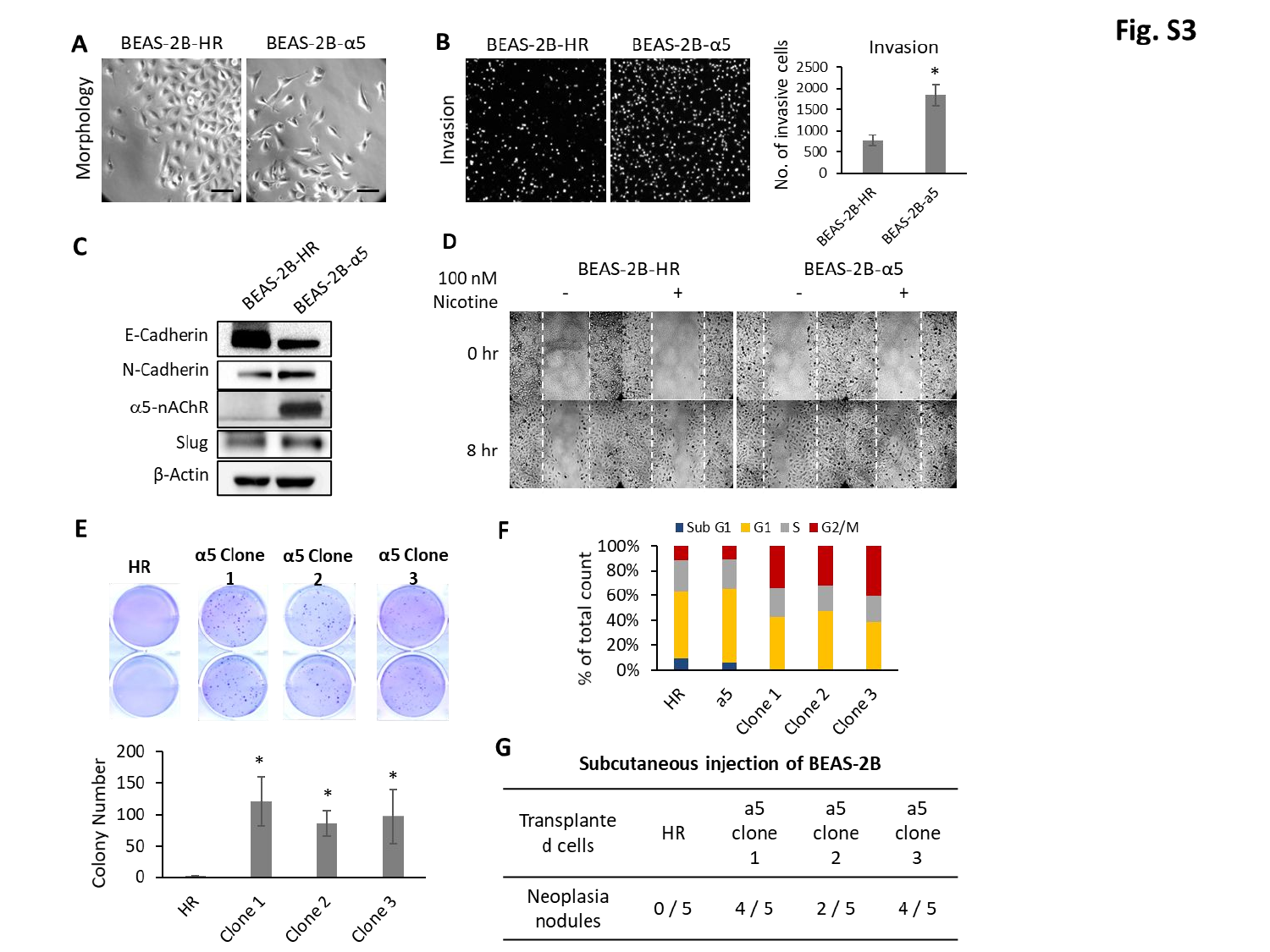

Supplement: Supplementary file 1 [file ijms-21-06829-s001.zip › Suppl Figure.pptx]
